# The Presenter’s Toolbox

**DOI:** 10.5195/jmla.2017.210

**Published:** 2017-07-01

**Authors:** Joy A. Russell

## INTRODUCTION

Most of us at some point in our careers need to present our ideas to an audience. We may present to library colleagues, potential employers, or students. We may provide training or present at conferences. In addition to tried-and-true tools like PowerPoint and Keynote, some online, web-native tools are now available. These tools are not only better suited to social sharing platforms than their desktop brethren but can be launched from any computer with a web browser—a huge relief during rare but inevitable hard-ware disasters. They also provide an opportunity to share presentations with others and to discover content that others have created.

All of the platforms discussed here offer free versions. But with all of the options available online, how do you begin to narrow your choices and select the right one for you? In this review, I share my first impressions of five online presentation services that will keep your audience engaged without tapping your wallet.

### SlideShare

SlideShare allows you to upload and share MS Word files, portable document format files (PDFs), presentations (PowerPoint, Keynote, or OpenOffice), infographics, video, and more from your desktop, Google Drive, Dropbox, or Gmail. Presentations can then be searched, viewed, and shared by anyone or embedded on other sites.

SlideShare is useful for medical librarians as a source of content, a source of example presentations to stimulate ideas and help you become a better presenter, and a platform for sharing presentations and building your professional brand. To get ideas or improve your skills in creating presentations, browse SlideShare to see what others are creating. The presentations featured on the SlideShare home page are a great place to start, as they have been handpicked by SlideShare and are likely to be good examples to follow.

You can also find presentations on topics related to yours, which may give you ideas to incorporate—with proper attribution—into your own work. SlideShare can also help you share your presentations with others and promote your professional brand. You can upload conference presentations, instructional slides, or other types of presentations to SlideShare.

Slideshare allows you to browse by category and create custom templates for presentations, so you can use a standard look and feel to make your presentations easily recognizable. Note that you will not be able to include narration, so your slides need to stand on their own without audio. To help people find your presentations, you can include up to twenty tags or keywords as well as a title and brief description of the presentation. Once you have uploaded your presentations, you can share the link with colleagues, share them via social media, and embed them in a website, blog, or LinkedIn profile.

At my institution, we have found that SlideShare is useful for facilitating teaching and learning on our campus through faculty, student, and staff research requests. Whether your client is preparing for a class project or grand round or is researching background information on a specific resource, SlideShare may have a ready-to-go presentation related to the topic.

### authorSTREAM

authorSTREAM is a free platform for uploading, hosting, and sharing PowerPoint, Keynote, and PDF presentations online. It has a large user base and helps you discover great presentations in various categories, including business and finance, education, product manuals, science, technology, and many others. As a presenter, author-STREAM makes it easy to share your slideshows through blogs, websites, and YouTube.

### Comparing authorSTREAM and SlideShare

Both Slideshare and authorSTREAM allow you to search and browse for presentations; determine whether or not to allow others to comment on or download your content; include videos in your presentations; determine an appropriate copyright or Creative Commons license for your content; share and embed content; view, like, comment on, follow, and often download the content of others; and capture analytics about number of views, downloads, comments, tweets, likes, and links.

authorSTREAM focuses on presentation files from PowerPoint and Keynote. It allows you to create channels, present live, and purchase templates from its Presentation Marketplace. A new feature offers you the ability to save presentations as videos for free as long as they are under five minutes. Unlike SlideShare, authorSTREAM allows you to include audio narration with your presentation. authorSTREAM also allows you to upload private or public presentations, even in a free account, unlike Slide-Share, which requires that all presentations up loaded with a free account be publicly available [1]. authorSTREAM also has my favorite feature, “present live,” which allows you to present in an online meeting in real time, while you provide your own audio.

I use both tools to follow other educators, other librarians, social media and marketing professionals, design professionals, and academics to see how they organize knowledge and make content visually engaging.

### Prezi

Prezi is different than both SlideShare and authorSTREAM. It is a virtual whiteboard for transforming presentations into compelling stories and visually flowing lessons. Using the zoomable interface, Prezi allows you to navigate from one idea to the next in whichever order you choose. As Prezi users will know, the order in which your presentation visits the various elements on the canvas is known as the “path.” When presenting, you can take things out of your path without taking them out of your presentation, so you can make a talk fit into a specific time length, but when people view the presentation afterward, they can see the full version with more detail.

Prezi is especially powerful when you want to make static content dynamic. You can stretch any image to any size, limited only by the resolution of the image. You can use an image as the background for your entire presentation and then add points of interaction with that image. It is also easy to embed YouTube videos: just copy and paste the uniform resource locator (URL), and Prezi does the rest. A presentation in Prezi can be shared on any platform and embedded into websites much like a YouTube video.

In addition to presentations, Prezi has other uses in academic libraries and is especially useful for presentations that focus on visual content. For example, you can create interactive maps of your library and/or specific areas, for example, to show the location of special collections or a department’s books and journals. I also use Prezi to create anatomy and physiology resource supplements. Prezi helps me make exploring the human body—even tedious step-by-step instructions—more engaging. You can embed a Prezi link in an online course to allow students and faculty to repeat lessons and access instructions anytime.

Prezi is also especially useful for presentations that cover several disparate subjects. When presenting on a single topic, the linear nature of PowerPoint works well. But when covering several topics, it can be helpful to show the audience all of them at first and then visit each of them, one by one. For example, in a session on online tools and technologies, the content is linked by little other than its format: online. Using Prezi can help attendees make sense of the broader context of these tools.

I find that Prezi encourages more engagement. People literally sit up in their seats and take notice, and they seem to remember the presentation more afterward. Whether you opt to fully customize a presentation from scratch or from one of the many available templates, Prezi’s approach is unique and allows you to take a more creative approach to presentations. It does, however, require some training to use.

### Projeqt

Projeqt allows you to create and share dynamic presentations from scratch or convert old static slides. Simply upload PowerPoint files, PDFs, or multiple image files at once and create a dynamic and portable online Projeq, with no coding required. Projeqts can be embedded and shared anywhere.

The tool also connects with a wide array of social media tools, allowing users to display live Twitter feeds, run Spotify playlists, reference Flickr images or Google maps, or incorporate a wealth of other live feeds, all updated in real time. A Projeqt presentation returned to in a week (or even an hour) may not be exactly the same as the one shared at a conference. To me, this feels as if Projeqt is inviting me to tell a story using social media tools. Seeing what the Projeqt community has created is a constant source of inspiration.

### emaze

emaze is another online presentation tool for professionals. With its zoomable interface that navigates from one idea to the next, emaze is similar to Prezi but differs in that you can present content three dimensionally. Unfortunately, emaze only allows new presentations to be created based on templates. That limits your ability to customize your own design, though emaze plans to allow custom designs soon in one of their premium plans. The program allows you to import existing PowerPoint slides. When you do, you will be prompted to apply an existing emaze theme to the presentation.

Like Prezi, emaze presentations are accessible everywhere via the cloud; can be downloaded as presentations, PDFs, or videos (with a premium plan); and can be embedded online. They also offer an offline viewer that you can use to view your presentation without an Internet connection.

emaze is a great product for library tutorials, introductions to class topics, and library video tours. They also offer a library of presentations that you can use and modify. While the free account is very good, paid accounts offer more features, and volume discounts are available for school districts and colleges.

Each of these five tools offers something different, but all of them are user friendly. If you are looking for a top-notch slide builder with an impressive, free option, any one of these products would be a great addition to your toolbox.
